# Exploring the Potential Impact of SERPINE Gene Expression in Cumulus Cells During Fertility Treatments: A Single Center Study

**DOI:** 10.3390/clinpract15050083

**Published:** 2025-04-23

**Authors:** Sofoklis Stavros, Anastasios Potiris, Despoina Mavrogianni, Efthalia Moustakli, Kyriaki Tsiorou, Athanasios Zikopoulos, Nikolaos Kathopoulis, Charalampos Theofanakis, Dimitrios Loutradis, Ekaterini Domali, Peter Drakakis

**Affiliations:** 1 Third Department of Obstetrics and Gynecology, University General Hospital “ATTIKON”, Medical School, National and Kapodistrian University of Athens, 124 62 Athens, Greece; sfstavrou@med.uoa.gr (S.S.); thanzik92@gmail.com (A.Z.); ctheofan@med.uoa.gr (C.T.); pdrakakis@med.uoa.gr (P.D.); 2 First Department of Obstetrics and Gynecology, Alexandra Hospital, Medical School, National and Kapodistrian University of Athens, 115 28 Athens, Greece; dmavrogianni@med.uoa.gr (D.M.); nickatho@gmail.com (N.K.); kdomali@yahoo.fr (E.D.); 3 Laboratory of Medical Genetics, Faculty of Medicine, School of Health Sciences, University of Ioannina, 451 10 Ioannina, Greece; ef.moustakli@uoi.gr; 4 Diagnostic and Therapeutic Fertility Institute S.A, 115 21 Athens, Greece; korinatsiorou@yahoo.com (K.T.); loutradi@otenet.gr (D.L.)

**Keywords:** cumulus cells, granulosa cells, assisted reproductive technologies (ARTs), infertility, SERPINE gene

## Abstract

**Background/Objectives**: Cumulus cells have been proposed to be indicators of oocyte quality. In this study, oocyte cumulus cells were analyzed for SERPINE gene expression. High SERPINE gene expression in cumulus cells is associated with reduced oocyte maturity. However, high mRNA levels in granulosa cells are associated with follicles that result in pregnancy. This study aimed to evaluate SERPINE gene expression in cumulus cells across different ovarian stimulation protocols and its potential impact on follicle number, oocyte maturity, and embryo quality. **Methods**: The sample of the study consisted of 93 infertile women that underwent a five-day fixed antagonist protocol. Detection of SERPINE gene expression levels in cumulus cells was performed by extracting and isolating the total RNA produced in granulosa cells, and conducting cDNA synthesis and Real-Time Polymerase Chain Reaction. **Results**: The SERPINE gene expression in CCs was assessed in 71 samples. The SERPINE gene expression levels in CCs were categorized based on the ΔCp values. Most participants (65.9%) exhibited a high expression of the SERPINE gene, with ΔCp values greater than 2. Higher gene expression resulted in a higher number of follicles. However, no statistically significant results were observed regarding the number of follicles and the number of embryos. **Conclusions**: The study results provide insights into the expression patterns of the SERPINE gene in CCs and underscore the complexity of fertility-related biomarkers and the need for further investigation. SERPINE expression appears to be associated with follicle count, while its role in predicting oocyte quality and pregnancy success remains inconclusive.

## 1. Introduction

Infertility is a common issue affecting one in six couples worldwide [1]. In vitro fertilization (IVF), which was first used in clinical settings in 1978, has completely changed human reproduction. In the beginning, it was developed to aid couples who suffered from infertility. Since then, IVF indications have expanded to include medical, genetic, and fertility preservation purposes [2]. The causes of infertility can be broken down into four main groups, including male factors, female factors, combined male and female factors, or unexplained etiology [3]. Despite recent advancements in assisted reproductive technology (ART), many patients still experience unexplained poor fertility outcomes. A better understanding of disease mechanisms and the introduction of novel biomarkers could potentially improve infertility diagnosis and predict pregnancy outcomes.

Cumulus cells (CCs) are the follicle cells surrounding the oocyte in cumulus–oocyte complexes, and their role in the developmental process of the oocyte is crucial [4]. CCs also support the maturation of oocytes via gap junctions [5]. Through these gap junctions, oocytes and CCs can communicate by utilizing paracrine and low-molecular-weight components [6]. Protein synthesis and gene expression patterns are influenced by these factors, which also promote CC differentiation [7]. Due to this critical crosstalk between the oocyte and these somatic cells during folliculogenesis, CCs and granulosa cells (GCs), have been proposed as oocyte quality markers by providing a deeper view of the microenvironment in which the oocyte acquires its developmental and reproductive competence [8]. GCs are also essential in the follicular differentiation process, which creates the ideal environment for oocyte development, ovulation, fertilization, and subsequent implantation. As a result, this last maturation may be used to determine the quality of the embryo [9]. Oocytes with a low chance of implantation are those whose maturation is not linked to CC expansion [10].

The ability of CC and GC gene expression profiles to function as prognostic biomarkers for oocyte and embryo competency as well as pregnancy has recently been investigated. The gene expression of CCs has been profiled in numerous studies to find gene markers and assess oocyte and embryo competency [11,12,13,14]. Proteases are essential for tissue remodeling and ovarian functions, including the considerable tissue remodeling which occurs during folliculogenesis and ovulation. They also play a major role in the proteolysis process. As an inhibitor of serine proteases, SERPINE controls proteolysis and contributes to implantation and folliculogenesis [15]. One of the proteins that inhibits serine proteases, from the serpin protein family, is encoded by SERPINE. This family member has the ability to inhibit several proteases, including plasmin, trypsin, urokinase, and thrombin. Serpins regulate coagulation and inflammation, among other biological processes, by protease inhibition, which makes these proteins a target for additional medical research [16,17]. One important biomarker in oncology is SERPINE, which is also overexpressed in several malignancies and aids in tumor growth. SERPINE mRNA levels in GCs have been suggested as a potential pregnancy biomarker [9]. It is mainly expressed in reproductive tissues, including the placenta, uterus, ovaries, and seminal vesicles [18,19]. Most studies indicate that high levels of this gene correlate with oocyte immaturity [20,21]. However, high levels of SERPINE mRNA in human GCs are associated with follicles that result in a successful pregnancy outcome [9].

This study evaluated SERPINE gene expression in CCs from infertile patients that had undergone ovarian stimulation. mRNA levels in CCs were assessed using real-time PCR. Ultimately, the aim of the present study was to assess the potential impact of SERPINE gene expression on oocyte and embryo quality.

## 2. Materials and Methods

### 2.1. Study Sample

The present study enrolled infertile couples who visited the Diagnostic and Therapeutic Fertility Institute S.A. in Athens, Greece, from April 2022 to June 2024 to undergo fertility treatment. Laboratory analyses were performed in the First Department of Obstetrics and Gynecology of the National and Kapodistrian University of Athens at Alexandra Hospital. The study included patients undergoing fertility treatments for tubal factor infertility, diminished ovarian reserves, and unexplained infertility. All participants had not received any hormonal therapy or ovarian stimulation for at least three months prior to the study. Couples with male infertility, autoimmune disorders, genetic disorders, chromosomal disorders, premature ovarian failure, endocrinological disorders, endometriosis, or other benign pathologies affecting fertility were excluded from the study.

The sample of the study consisted of 93 infertile couples. For each participant, the following data were collected: age, duration of infertility, number of previous ART cycles, cause of infertility, body weight, height, and body mass index (BMI). Table 1 provides the demographic characteristics of the study sample.

### 2.2. Ethical Approval and Patient Consent

The study was approved by the Ethics Committee of the Diagnostic and Therapeutic Fertility Institute S.A. with the identifier 10/20.12.2020 and the Institutional Review Board of the Medical School of the National and Kapodistrian University of Athens with the protocol identifier 125252/30 November 2023. Participation in the study was voluntary, and all couples provided written informed consent before the initiation of the assisted reproductive technique cycle. Participation in the study only included obtaining the approval to assess the SERPINE gene expression. The protocol selection, the initiation dose of gonadotropins, and the selection of the gonadotropin regimen were independent from the participation.

### 2.3. Controlled Ovarian Stimulation

All women in the present study underwent controlled ovarian stimulation following GnRH antagonist protocol. On day two of the cycle, gonadotropin therapy was initiated at a dose of 200 IU, with minor adjustments based on ovarian response. The antagonist was initiated on day five (fixed antagonist protocol) of the stimulation. The regimen used for all women was ganirelix (Orgalutran, MSD Hellas).

From day 7 of the cycle, serum estradiol (E2) levels were monitored daily, and follicular growth was assessed via daily ultrasound scans. The final triggering of maturation was initiated when the cohort of follicles reached ≥18 mm in diameter and E2 levels exceeded 600 pg/mL. Oocyte retrieval was performed 36 h after triggering under intravenous anesthesia. Following oocyte retrieval, IVF and ICSI procedures were performed. Embryo quality was assessed based on blastomere count, degree of fragmentation, blastomere consistency, and multinucleation.

### 2.4. Cumulus Cell Selection

At the end of oocyte retrieval, CCs were maintained in a suitable culture medium inside an incubation oven under controlled temperature, humidity, and CO_2_ conditions for four hours. During this period, the oocyte extruded the first polar body, being designated as an MII oocyte ready for fertilization. After four hours, the culture plate containing the oocytes was removed from the incubator.

Under a microscope, oocytes were enzymatically treated with hyaluronidase to remove GCs before fertilization. The isolated GC samples were collected using a specialized glass pipette, transferred into eppendorf tubes, and stored at −80 °C until further analysis. Each sample was then coded with a unique identifier (1–93), and the experimental protocol was followed. To determine SERPINE gene expression levels in CCs, the total RNA was extracted and isolated, followed by cDNA synthesis and Real-Time Polymerase Chain Reaction (RT-PCR) analysis.

### 2.5. Isolation of RNA (Extraction Process)

After collecting all the CCs, the samples were centrifuged at 14,000 rpm for 1 min to achieve cell lysis and homogenization. Then, 300 μL of RNA Lysis Buffer was added to the samples, and the solution was transferred to a DNA Removal Column (light blue) fitted with a collection tube. This column retained the unwanted products from cell lysis (proteins, phospholipids). The column was then centrifuged at 14,000 rpm for 2 min.

The DNA Removal Column was discarded, and 300 μL of 95% ethanol was added to the solution that had passed through the filter. The solution was gently pipetted 4–5 times to ensure a thorough mix. The addition of ethanol created suitable conditions for RNA binding to the RNA purification column.

The solution was transferred to an RNA Purification Column (dark blue) with an RNA-binding filter and centrifuged at 14,000 rpm for 2 min. The flow-through was discarded, and the filter was replaced into the column. Then, 500 μL of RNA Priming Buffer was added, followed by centrifugation at 14,000 rpm for 2 min.

The flow-through was discarded again, and the filter was placed back in the same column. Next, 500 μL of RNA Wash Buffer was added, and the column was centrifuged at 14,000 rpm for 2 min. This wash step was repeated with an additional 500 μL of RNA Wash Buffer, and the column was centrifuged at 14,000 rpm for 4 min.

The filter was transferred to a specialized eppendorf tube, and 30 μL of nuclease-free water was added to elute the RNA. The solution was centrifuged at 14,000 rpm for 2 min. Finally, the filter was discarded, and the purified RNA was collected in the eppendorf tube and stored at −80 °C.

### 2.6. Complementary DNA (cDNA) Strand Synthesis

The isolated RNA, previously stored at −80 °C, was placed in a cooler for gradual thawing. Simultaneously, the preparation for cDNA synthesis was initiated. First, the sample was transferred into 0.5 mL eppendorf microtubes. A reaction mix was prepared in a separate 0.5 mL eppendorf microtube containing LunaScript RT SuperMix 4x (for the number of samples + 1) and nuclease-free water (for the number of samples + 1) μL. To each microtube, 15 μL of the reaction mix and 5 μL of the corresponding RNA sample were added. The microtubes were then transferred to a thermocycler, where they were incubated at 25 °C for 10 min. Following incubation, the microtubes were stored at −20 °C for future use.

### 2.7. Real-Time Polymerase Chain Reaction (Real-Time PCR)

Quantitative PCR (Real-Time qPCR) using the two-step method was employed in the present study with the LightCycler 480 Real-Time PCR Instrument (Roche, Basel, Switzerland). Two separate 0.5 μL eppendorf microtubes were prepared, each containing a mix solution for the SERPINE and G6PD genes, with primers designed to have a melting temperature (Tm) of 60 °C for optimal binding efficiency. G6PD was used as the reference gene for comparison.Table 2 summarizes the reagents and primer pair sequences used for this study.

For each sample, 20 μL of the prepared mix and 5 μL of the cDNA were added to a special microplate. Samples of SERPINE were placed in one row, and the corresponding G6PD samples were placed in the columns. The microplate was covered with an optically transparent film and placed into the LightCycler 480 Real-Time PCR Instrument.

Cp (Crossing Point) values for both SERPINE and G6PD were recorded. Cp values are inversely proportional to the gene copy number: a higher gene expression results in a lower Cp value. To calculate the SERPINE gene expression level in CCs, the ΔCp value was determined by subtracting the Cp for G6PD from the Cp for SERPINE for each sample. A ΔCp value greater than 2 was considered a high expression, while a ΔCp value less than 2 indicated a low expression.

## 3. Results

### 3.1. Controlled Ovarian Stimulation, Oocytes, and Embryos

From the 93 ovarian stimulations, 60.9% of women had six-to-ten follicles visible on the ultrasound, 3.3% had fewer than five follicles, and 35.9% had more than ten follicles. The mean number of mature oocytes was approximately eight (SD = 2.6), and the mean number of fertilized eggs was seven (SD = 2.5). Regarding embryo quality, most women (75%) had good-quality embryos, 20.7% had excellent-quality embryos, and the remaining 4.3% had poor-quality embryos. Table 3 provides the compound outcomes from the ovarian stimulation of the sample.

ANOVA assumptions were met, including normality (assessed by the Kolmogorov–Smirnov test), homoscedasticity (assessed by Levene’s test), and the independence of observations. These assumptions are crucial for ensuring the validity of ANOVA results, as violations can lead to biased conclusions. Normality ensures that residuals follow a Gaussian distribution, homoscedasticity maintains equal variances across groups, and independence prevents data points from influencing each other. Following verification, ANOVA was conducted to compare the four different ovarian stimulation regimens, namely rFSH, rFSH + HCG, hMG, and hMG + HCG in relation to the number of follicles (*p* < 0.001), mature oocytes (*p* < 0.001), and fertilized oocytes. The significance level was set at 5% (α = 0.05). The rFSH protocol yielded the highest number of follicles, mature oocytes, and fertilized oocytes, while the hMG + hCG protocol resulted in the lowest values, with significant differences compared to the other groups. However, the samples were relatively small for extrapolating results based on the regimen used in the fixed antagonist protocol. Furthermore, it was not within the scope of the present study to compare the different regimens, only the expression of the SERPINE gene. Hence, all the results are presented according to the ΔCp values as explained in Section 3.2.

### 3.2. SERPINE Gene Expression

The SERPINE gene expression in CCs was only able to be assessed in 71 samples due to the inadequate CC samples; no ovarian stimulation and inconsistent extreme values were found in the remaining 22 samples. The SERPINE gene expression levels in CCs were categorized based on the ΔCp values. Most participants (65.9%) exhibited a high expression of the SERPINE gene, with ΔCp values greater than 2. In contrast, 34.1% of the participants showed low expression of the SERPINE gene, with ΔCp values less than 2. These results are summarized in Table 4.

Regarding gene expression and the number of follicles observed, our study results showed that higher ΔCp expression was associated with a higher frequency of follicles being observed in both the five-to-ten and over ten follicle subgroups. However, this association did not meet statistical significance. Table 5 presents the number of follicles observed in both expression subgroups.

Lastly, regarding embryo quality, there was no significant association observed. In this analysis, the higher gene expression subgroup had a higher frequency in both excellent- and good-quality embryos, but there was no statistically significant association. Table 6 represents the study results regarding SERPINE gene expression and embryo quality.

## 4. Discussion

Gene expression signatures in CCs have recently been investigated for their potential application as prognostic biomarkers for pregnancy outcomes and oocyte and embryo competence [13,14]. CCs provide a non-invasive way to evaluate oocyte maturity, embryo quality, and, in turn, the chance of becoming pregnant, because they are eliminated throughout the IVF procedure [4,13].

To ascertain the degree of SERPINE gene expression, CCs were taken from infertile women undergoing IVF and were examined. One possible biomarker for evaluating the developmental ability of oocytes is SERPINE. According to recent research, CCs with high SERPINE gene expression have a lower oocyte maturity [20,21], whereas those with low SERPINE expression have a higher oocyte maturity [22]. Oocyte maturation was drastically reduced by either the exogenous addition of SERPINE or its overexpression in mice GCs. With a larger ratio of GV to MI oocytes and a compact shape, GCs demonstrated decreased egg maturation. The idea that excessively high SERPINE levels correspond with oocyte immaturity may be supported by these data, which may explain why cells surrounding immature human oocytes express higher levels of SERPINE than those surrounding mature eggs [21]. Nevertheless, some research has also demonstrated that follicles that result in successful pregnancies are linked to high levels of SERPINE gene expression in CCs [9,18].

The majority of participants (65%) in our study showed significant SERPINE gene expression in CCs, which was one of our main findings. However, the expression of the SERPINE gene was not significantly associated with the number of follicles, the number of mature oocytes, and embryo quality according to our statistical study. This finding is particularly important because it suggests that the difference in SERPINE expression may not directly influence oocyte developmental potential or embryo implantation success [23]. These results align with prior studies indicating that the SERPINE gene is involved in ovarian tissue remodeling and follicle development but does not necessarily dictate oocyte competency. Evidence suggests that SERPINE overexpression may affect follicular growth by altering the dynamics of the extracellular matrix instead of directly affecting oocyte maturation [24,25,26]. This emphasizes the necessity of investigating whether the expression of the SERPINE gene mirrors follicular activity or acts as a trustworthy biomarker for reproductive outcomes. The main goal of future studies should be to ascertain if SERPINE expression is a direct result of ovarian stimulation methods or a measure of oocyte quality.

Given that ovarian stimulation protocols impact follicular development and hormonal microenvironments, further research is needed to determine whether changes in gene expression are a direct result of protocol selection or if they reflect inherent biological variability among patients [22,27]. Additionally, assessing the longitudinal expression of SERPINE throughout the IVF cycle could provide more insight into its role in follicular maturation and embryo competence [25].

While we observed a higher follicle count in the high-expression group, it is important to note that this study did not establish a causal relationship between SERPINE and oocyte viability. The absence of a statistically significant correlation between SERPINE expression and fertility outcomes suggests that additional molecular markers should be considered when assessing embryo quality and implantation potential. Future research should explore whether a combination of multiple gene expression profiles in CCs could serve as a more robust predictor of IVF success.

Furthermore, our findings highlight the necessity of large-scale, multicenter studies to validate SERPINE as a potential biomarker in reproductive medicine. Given the relatively small sample size of our study (n = 93), the statistical power may have been limited in detecting subtle but clinically significant associations. Additionally, as a single-center study, site-specific factors such as the inclusion of mainly Caucasian women may have influenced the results. Future research with larger, multicenter cohorts and longitudinal follow-up is needed to further clarify these findings. Moreover, a further division of the different subgroups based on the regimen used for controlled ovarian stimulation or based on the cause of infertility would also be an interesting aspect for further research. Additional studies incorporating transcriptomic and proteomic analyses could further clarify the role of SERPINE in reproductive physiology and its potential implications for fertility treatments. More analytically, the regulation and downstream signaling of the SERPINE gene should be extensively studied, including the effect of miRNAs in regulating its expression, and the methylation sites of the SERPINE sequence which encode the corresponding protein and eventually influence its expression. The detection of single nucleotide polymorphisms and/or mutations of the genomic sequence of the SERPINE gene and their relationship with the action of the corresponding protein may also be a future research project. Finally, the application of siRNA techniques to reveal the molecular and cellular consequences of the absence of the gene in oocytes may show the importance of the presence of the gene.

Consequently, our study provides valuable insights into the expression patterns of the SERPINE gene in CCs and its potential implications for folliculogenesis and embryo development. However, our findings also underscore the complexity of fertility-related biomarkers and the need for further investigation. While SERPINE expression appears to be associated with follicle count, its role in predicting oocyte quality and pregnancy success remains inconclusive. Future research should aim to integrate genetic, epigenetic, and metabolic profiling to enhance our understanding of the molecular mechanisms governing oocyte competence and embryo viability. Such knowledge could ultimately aid fertility specialists in optimizing treatment strategies and improving ART outcomes for patients.

## 5. Conclusions

The importance of proper follicular development is vital for maintaining fertility in humans. There is a significant gap in the literature regarding the understanding of the specific mechanisms involved during the progression of ovulation, highlighting the complexity and the multitude of factors that regulate the entire process. Our study showed that a higher SERPINE gene expression may result in a higher number of follicles in ovarian stimulation, but no significant association was observed. Further study of the SERPINE gene could provide a valuable tool for reproductive profiling. Additionally, investigating this gene in CCs, which reflect the condition of the oocyte, could offer a molecular non-invasive means of evaluating both follicle and oocyte competence. Hence, it is essential to conduct further research to determine the gene expression in GCs and to identify those genes that could ultimately affect oocyte maturity and, consequently, human reproduction. Having reliable indicators would assist fertility specialists in selecting the most appropriate treatment approach, thus reducing failure rates, costs, and the physical and psychological strain on couples.

## Figures and Tables

**Table 1:** Demographic characteristics of the study sample (n = 93).

Variable	Category	Frequency	Percentage (%)
Age (years)	<30	5	5.4
	30–40	69	74.2
	>40	19	20.4
BMI (kg/m^2^)	<20	14	15.2
	20–25	61	65.6
	25–30	11	11.8
	>30	7	7.4
Years of Infertility	0–5	67	72.0
	6–10	19	20.4
	>10	7	7.6
Previous ART attempts	0–2	83	89.2
	3–4	8	8.6
	>4	2	2.2
Reason for ART	Tubal Factor	12	12.9
	Unexplained Infertility (Including Failed IUIs)	51	54.8
	Diminished Ovarian Reserves (DOR)	30	32.3

BMI: body mass index, ART: assisted reproductive technology, IUI: intrauterine insemination, DOR: diminished ovarian reserves.

**Table 2:** Reagents and primer sequences.

Component	Concentration
KAPA SYBR qPCR Master Mix	12.5x (samples + 1)
Nuclease-free water	5.5x (samples +1)
Primers for SERPINE	1:10 dilution
Primers for G6PD	1:10 dilution
SERPINE primer pair	
Primer type	Sequence
Forward (F)	5′-TCT CAT TGC AAG ATC ATC GCC-3′
Reverse (R)	5′-CCC CAT GAA TAA CAC AGC ACC-3′
G6PD primer pair	
Primer type	Sequence
Forward (F)	5′-TGG ACC TGA CCT ACG GCA ACA GAT A-3′
Reverse (R)	5′-GCC CTC ATA CTG GAA ACC C-3′

**Table 3:** ART outcomes in the study sample.

Demographic Variable	Category	Frequency	Percentage (%)
Number of Follicles (>12 mm)	<5	3	3.3
	6–10	56	60.9
	>10	33	35.9
Embryo Quality	Excellent	19	20.7
	Good	69	75.0
	Poor	4	4.3

**Table 4:** SERPINE gene expression.

Expression of SERPINE	ΔCp	Frequency	Percentage (%)
High	>2	46	64.79
Low	<2	25	35.21

**Table 5:** SERPINE gene expression and number of follicles observed in ovarian stimulation.

			Number of Follicles (>12 mm)			Total
			<5	5–10	>10	
ΔCp cutoff	ΔCp < 2	Count	1	18	6	25
		%	4.0%	72.0%	24.0%	100.0%
	ΔCp > 2	Count	0	29	17	46
		%	0.0%	63.0%	37.0%	100.0%
Total		Count	1	47	23	71
		%	1.4%	66.2%	32.4%	100.0%
					*p* value	0.237

**Table 6:** SERPINE gene expression and embryo quality.

			Embryo Quality			Total
			Excellent	Good	Poor	
ΔCp cutoff	ΔCp < 2	Count	4	20	1	25
		%	16.0%	80.0%	4.0%	100.0%
	ΔCp > 2	Count	12	32	2	46
		%	26.1%	69.6%	4.3%	100.0%
Total		Count	16	52	3	71
		%	22.5%	73.2%	4.2%	100.0%
					*p* value	0.614

## Data Availability

The raw data supporting the conclusions of this article will be made available by the corresponding author on request.
